# The synergistic effects of saxagliptin and metformin on CD34+ endothelial progenitor cells in early type 2 diabetes patients: a randomized clinical trial

**DOI:** 10.1186/s12933-018-0709-9

**Published:** 2018-05-03

**Authors:** Fiona J. Dore, Cleyton C. Domingues, Neeki Ahmadi, Nabanita Kundu, Yana Kropotova, Sara Houston, Carol Rouphael, Aytan Mammadova, Linda Witkin, Anamil Khiyami, Richard L. Amdur, Sabyasachi Sen

**Affiliations:** 1 The GW Medical Faculty Associates, Washington, DC, USA; 2 Department of Medicine, The George Washington University, 2300 Eye Street, Washington, DC, 20037 USA; 3 Princess Nora Bint Abdul Rahman University, Riyadh, Saudi Arabia; 4 Present Address: Department of Internal Medicine, Cleveland Clinic, Cleveland, OH USA; 5 Present Address: Richmond University Medical Center, Staten Island, NY USA; 6 Present Address: Weill Cornell Medicine/New York7-Presbyterian, New York, USA

**Keywords:** Diabetes, Endothelial progenitor cells, Saxagliptin, DPP-4 inhibitor, Arterial stiffness

## Abstract

**Aims:**

Type 2 diabetes is associated with endothelial dysfunction leading to cardiovascular disease. CD34+ endothelial Progenitor Cells (EPCs) are responsible for endothelial repair and neo-angiogenesis and can be used as a cardiovascular disease risk biomarker. This study investigated whether the addition of saxagliptin, a DPP-IV inhibitor, to metformin, may reduce cardiovascular disease risk in addition to improving glycemic control in Type 2 diabetes patients.

**Methods:**

In 12 week, double-blind, randomized placebo-controlled trial, 42 subjects already taking metformin 1–2 grams/day were randomized to placebo or saxagliptin 5 mg. Subjects aged 40–70 years with diabetes for < 10 years, with no known cardiovascular disease, BMI 25–39.9, HbA1C 6–9% were included. We evaluated EPCs number, function, surface markers and gene expression, in addition to arterial stiffness, blood biochemistries, resting energy expenditure, and body composition parameters. A mixed model regression to examine saxagliptin vs placebo, accounting for within-subject autocorrelation, was done with SAS (p < 0.05).

**Results:**

Although there was no significant increase in CD34+ cell number, CD31+ cells percentage increased. Saxagliptin increased migration (in response to SDF1α) with a trend of higher colony formation count. MNCs cytometry showed higher percentage of CXCR4 double positivity for both CD34 and CD31 positive cells, indicating a functional improvement. Gene expression analysis showed an upregulation in CD34+ cells for antioxidant SOD1 (p < 0.05) and a downregulation in CD34− cells for IL-6 (p < 0.01). For arterial stiffness, both augmentation index and systolic blood pressure measures went down in saxagliptin subjects (p < 0.05).

**Conclusion:**

Saxagliptin, in combination with metformin, can help improve endothelial dysfunction in early diabetes before macrovascular complications appear.

*Trial registration* Trial is registered under clinicaltrials.gov, NCT02024477

**Electronic supplementary material:**

The online version of this article (10.1186/s12933-018-0709-9) contains supplementary material, which is available to authorized users.

## Background

Type 2 diabetes is a national epidemic, affecting 11% of adults in the United States [1, 2]. Both diabetes and prediabetes are associated with significant macro and microvascular complications, including endothelial dysfunction, oxidative stress, endothelial cell inflammation, cardiovascular pro-thrombotic states, and kidney disease [1, 3–5]. Therefore, endothelial progenitor cells (EPCs, defined here as CD34+ cells), which are specialized cells responsible for endothelial repair and neo-angiogenesis, play an important role in diabetes. It has been shown that EPCs are impaired in number, function and gene expression in hyperglycemia and diabetes related complications [6–10]. Moreover, it has been reported that EPCs (CD34+) from diabetic patients failed to incorporate and repair damaged vessels [11]. EPCs can act as a cellular biomarker that is more reliable than serum based markers for estimating and following endothelial dysfunction in early type 2 diabetes patients. Thus, investigating EPCs could help develop a cardiovascular disease (CVD) risk estimation [12–15].

Dipeptidyl peptidase-4 (DPP-4) inhibitors, a popular class of anti-diabetic medications, have been shown to achieve improved glycemic control by lowering HbA1C, without causing hypoglycemia, and are weight neutral [16]. Because DPP-4 degrades particular incretins, such as SDF-1ɑ, its inhibition is also linked with a potential mechanism to prevent vascular diseases. However, there is limited data demonstrating the potential cardiovascular effects of these medications. Only a few studies using either sitagliptin or saxagliptin have shown an increase in endothelial progenitor cells, and thus potential cardiovascular benefits, with DPP-4 therapy [12, 13, 17].

Metformin has commonly been used as the first line pharmacologic agent for treating diabetes and pre-diabetes as per the American Diabetes Association guidelines [18]. Metformin improves glycemic control by decreasing hepatic glucose production, decreasing glucose absorption in the intestines and stomach, and increasing insulin-mediated glucose uptake [19]. Metformin has shown cardio-protective effects by increasing endothelial progenitor cells and CFU-Hill’s colonies in type 1 diabetes, and is known to also have cardio-protective properties in type 2 diabetes [20–22].

The up-regulation of SDF-1α and vascular endothelial growth factor (VEGF), both chemotactic factors, in serum increases mobilization and recruitment of EPCs in the face of acute ischemic injury for repair and regeneration [23–26]. It is postulated that DPP-4 inhibitors may increase EPCs mobilization from the bone marrow via their role in increased SDF-1α presence in serum [12].

Since poor viability and impaired function of EPCs in early diabetes will ultimately affect the repair and regeneration of the endothelium, a prompt intervention may help to reduce or reverse cardiovascular risk by improving EPCs survival and function above and beyond adequate glucose metabolism control. In this 12 week placebo-controlled clinical trial, we investigated the effect of saxagliptin, a DPP-4 inhibitor, in addition to metformin and exercise, on endothelial dysfunction in early type 2 diabetes patients who do not have any established macro-vascular complications.

## Methods

This Phase 4, single-site, double-blind, placebo-controlled, randomized clinical trial was approved by The George Washington University Institutional Review Board, and was conducted in accordance with Good Clinical Practices of the National Institutes of Health.

Data were analyzed in accordance to the pre-determined statistical plan. To minimize potential bias, the study team, in addition to the research subjects, remained blinded to each subject’s randomized group, until every subject had finished the research study, and all data had been compiled, locked, and analyzed. Un-blinding was performed by the study statistician 6 months after all subjects had completed the study.

### Participants

42 adults with Type 2 Diabetes diagnosed within 10 years, currently on metformin (1000–2000 mg/day) were enrolled. Subjects were between 40 and 70 years of age, with a BMI of 25–39.9 kg/m^2^, and a HbA1C between 6.0 and 9.0%. Additional inclusion/exclusion criteria can be found in Additional file 1: Appendix S1. This study consisted of a single site at The GW Medical Faculty Associates.

### Study design and treatment

Once subjects signed the informed consent, and were found eligible, there was a 1 month “wash in” period, during which subjects adjusted their exercise level in order to achieve 150 min of moderate-intensity physical activity per week. Diet counseling was also provided. At visit 1, baseline values of the following measures were gathered: blood biochemistries, vitals, biophysical parameters, resting energy expenditure (REE), arterial stiffness measures, and endothelial progenitor cells analysis. Subjects were then randomized to one of two arms: saxagliptin 5 mg/day or placebo, in a blinded manner. Subjects took either saxagliptin (n = 21) or placebo (n = 21) for 12 weeks, while engaging in 150 min of moderate intensity physical activity per week. Visits were conducted every 6 weeks, ending at week 12 (visit 3).

### Endothelial progenitor cells analysis

Peripheral blood samples (approximately 60 ml) were drawn from patients and diluted in phosphate buffered saline (1:1). Mononuclear cells (MNCs) were then isolated from whole blood using a Ficoll density centrifugation method. MNCs were counted and an aliquot was used for CFU-Hill colony formation assay following the manufacturer’s instructions (Stem Cell Technologies, Vancouver, BC, Canada). At day 5 colony forming units (CFU) were counted. A fraction of MNCs were stained with (FITC, PE, APC)-conjugated antibodies (Miltenyi Biotec GmbH, Bergisch Gladbach, Germany) in order to analyze specific endothelial cell surface markers (CD34, CD31, CXCR4) by flow cytometry.

To isolate EPCs (CD34+), MNCs were magnetically sorted through a column after cells were stained with CD34 microbeads antibody (Miltenyi Biotec GmbH, Bergisch Gladbach, Germany). According to the manufacturer and based on flow cytometry analysis, the purity of CD34+ cells post sorting is 67% (before gating on white blood cells). An aliquot of CD34+ cells were then stained with trypan blue and counted using an Auto Cellometer Mini (Nexcelom Bioscience, Lawrence, MA).

CD34+ gene expression analysis was performed by quantitative reverse transcriptase polymerase chain reaction (qRT-PCR). CD34+ total mRNA was extracted and purified using the RNeasy mini kit (Qiagen). mRNA was then converted into cDNA by using the high capacity cDNA reverse transcription kit (Applied Biosystems). Possible gene expression changes promoted by Saxagliptin were assessed by a CFX96 real-time qPCR system (Bio-Rad) using TaqMan Universal Master Mix II (Applied Biosystems) and inventoried probes. The gene expression analysis included antioxidants, apoptosis, endothelial function, chemotaxis, inflammation, and endothelial lineage cell surface markers. The expression of individual gene was normalized to either housekeeping 18S or GAPDH and calculated by using the 2^−∆∆Ct^ method considering the difference in cycle threshold between visit 2 or visit 3 and baseline (visit 1). Gene expression of CD34− cell population was also analyzed along with CD34+ cells.

The migratory capacity of CD34+ was evaluated using the CytoSelect 24-well Cell Migration Assay kit (Cell Biolabs, Inc., San Diego, CA). Cells were suspended in serum-free media and seeded at 100,000 cells per insert. Migration of the cells through a 3 µm polycarbonate membrane to the wells containing serum-free media (control) and chemoattractant SDF-1α (10 or 100 ng/mL) was assessed after cells were kept overnight in a CO_2_ incubator at 37 °C. Migratory cells were dissociated from the membrane and subsequently lysed and quantified by fluorescence (480 nm/530 nm) using CyQuant GR dye (Cell Biolabs, Inc., San Diego, CA). The fluorescence ratios between cells exposed to the chemotactic factor and cells exposed to chemoattractant-free media (control) along the visits were used to analyze the migratory capacity of the cells.

### Clinical and laboratory measures

Arterial stiffness was assessed through pulse wave analysis (PWA), and pulse wave velocity (PWV). PWA was obtained from the radial artery while the subjects were seated at rest. Investigators tried to obtain a minimum of three measures, with an operator index score ≥ 80. PWA measures include: augmentation index (AI), Augmentation Index adjusted for a heart rate of 75 (AI-75), augmentation pressure (AP), and both systolic and diastolic blood pressures (SBP, DBP) measured both centrally and peripherally. PWV measures the velocity of the pulse as it moves from a “proximal” artery to a “distal” artery. The designated proximal artery was the carotid, however, occasionally the radial artery was used if no carotid measurement could be obtained. The designated distal artery was the femoral artery, with no alternative used. PWV was obtained with subjects supine, at rest. Investigators tried to obtain a minimum of two measures, each with a standard deviation of less than 10%. These measured were gathered using the AtCor SphygmoCor CP system.

Basal metabolic rate, otherwise known as resting energy expenditure (REE), was measured using the KORR REEVUE. Subjects were resting, sitting in an exam chair prior to beginning the test. Tests ran between 10 and 15 min. Values gathered include: Measured REE, Predicted REE, estimated total energy expenditure, VO2, and calories per day.

Body composition parameters were gathered both manually and using a Tanita BF-350 body composition scale. Manual measurements include: height, weight, BMI, waist circumference, hip circumference. The Tanita scale works via bio-impedance, and provides measures on: weight, BMI, percent body fat, fat mass (kg), fat free mass (kg), percent body water, water mass (kg), basal metabolic rate (kcal), daily calorie intake (kcal), and impedance.

A venous blood draw was performed for both biochemical analyses and serum ELISA. Standard of care laboratory measures were collected at each visit to monitor trends and changes. The following values were ordered either as plasma, serum, or whole blood through Laboratory Corporation of America: basic metabolic panel, lipid panel, leptin, HbA1C, C-reactive protein, IL-6, Adiponectin, and Insulin. ELISA was performed in order to analyze serum total GLP1 and SDF-1α. GLP-1 was analyzed using a competitive ELISA Immunoassay Kit (Raybiotech, Norcross, GA), and SDF-1α using a sandwich ELISA (EHCXCL12A, Thermo Scientific).

Vitals gathered were congruent with those gathered as a part of standard of care: Resting blood pressure, pulse, and temperature.

Finally, subject’s level of exercise exertion was measured using Actigraph wGT3X-BT activity monitors. Subjects were instructed to wear the Actigraphs during all waking hours for a total of 7 consecutive days. Subjects were provided dietary and exercise advice, as a part of the study. For exercise, all subjects were instructed at screening to achieve 150 min/week of moderate intensity physical activity, as per the ADA guidelines. Actigraphs served as a measure of this exercise compliance, and to verify for exercise as a confounding variable.

### Statistical analyses

Variable distributions were examined using frequency histograms and outliers were excluded. In gene expression variables, outliers with expression values > 50 were dropped, and values were natural-log transformed due to skewness [using log(expression + 1)]. Expression values that still had outliers after log transformation were capped at a value of 2.

For each dependent variable (dv), we used a random effects mixed model with robust standard errors to estimate the group (saxagliptin/placebo) main effect, the visit (1, 2, 3) main effect, and the group x visit interaction. The main effect of group tells whether the saxagliptin patients vs controls differed on the dv independently of visit. The visit main effect tells us whether the dv mean changed across visits, independently of treatment group. The treatment x visit interaction was of primary interest, since this tells us whether the treatment and control groups had different slopes over visits, i.e. whether the pattern of scores over time differed based on treatment. Because there was a slight change in the laboratory procedure for CFU and migration outcome measurements after subject 20, we also considered this variation and included 3-way interaction tests (group x visit x early/late) in the model for these parameters. Since subjects were randomized to treatment, baseline subject variables that differed between treatments could not act as confounds (i.e. could not cause a spurious association between treatment and outcome, because they could not affect treatment assignment). Therefore, randomized controlled trials do not usually adjust for baseline differences. However, as a sensitivity analysis, for dependent variables with significant effects, we included as a covariate in the mixed model, any baseline variable that differed between treatment groups with p < 0.10.

SAS (Version 9.3 or 9.4, Cary, NC) was used for data analysis with p < 0.05 considered significant.

## Results

Patient disposition was similar between saxagliptin and placebo groups (Table 1), with no statistical significance at baseline. There was a trend-level difference noted in HbA1C (p = 0.06), so it was controlled for in sensitivity analysis. There was no significant difference in attrition at visits 2 or 3 between groups.

**Table 1:** Baseline demographics and disease characteristics

	Saxagliptin*	Control*
*N*	21	21
Age (years), mean ± SD	58.3 ± 5.7	56.4 ± 8.5
Female, n (%)	11 (52%)	7 (33%)
Race, n (%)		
African American	15 (71%)	13 (62%)
White	5 (24%)	6 (29%)
Other	1 (5%)	2 (10%)
Weight (lbs), mean ± SD	202.7 ± 24.5	202.3 ± 38.7
BMI (kg/m^2^), mean ± SD	32.3 ± 4.2	31.5 ± 4.8
Duration of diabetes (years), mean ± SD	3.7 ± 2.4	3.5 ± 1.8
HbA1C mean ± SD	7.0 ± 0.8	6.6 ± 0.5
Fasting glucose (mg/dL), mean ± SD	127.4 ± 35.9	114.8 ± 25.0
eGFR (mL/min/1.73), mean ± SD	98.9 ± 14.5	93.7 ± 16.7

* No significant differences were observed

### Primary outcomes: cellular

#### CFU-Hill’s colonies

**Fig. 1:**
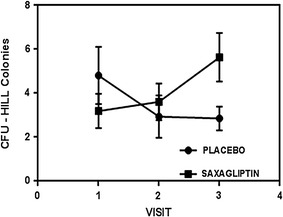
CFU-Hill’s colonies as an indicator of vascular health. Experiments were performed in duplicate and values are given as mean ± SD (p = 0.07, for the visit x treatment interaction t-test in a random effects mixed model)

The CFU-Hill’s colonies formation was improved in the saxagliptin treatment group. The ascendant curve in Fig. 1 shows that CFU numbers increased across 12 weeks (visit 1–3) of saxagliptin treatment, while a decline in the curve was found in the placebo group. Despite these results being close to statistical significance (p = 0.07), they clearly indicate a positive effect of the drug on the functionality of the endothelial cell lineage, and the lack of full significance is likely due to a small sample size.

#### Endothelial progenitor cells (CD34+)

**Fig. 2:**
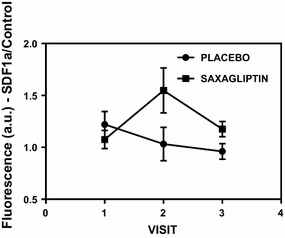
Migration of CD34+ cells in response to SDF-1α (100 ng/mL). Results are expressed as fluorescence ratio between cells exposed to the chemotactic factor and cells exposed to chemoattractant-free media (control) followed by lysis in presence of CyQuant GR dye. Experiments were performed in duplicate and results are given as mean ± SD (p < 0.05)

The percentage of CD34+ cells purified from the MNCs population did not show any significant difference neither at baseline nor along the study between treatment and control groups. Although EPCs (CD34+) number did not differ by treatment group (2.8% ± 0.5 for saxagliptin group vs 2.0% ± 0.3 for placebo group), the migratory response of CD34^+^ cells to the chemotactic factor SDF-1α (100 ng/mL) was significantly greater (p = 0.04). This functional improvement can be better appreciated at visit 2 (Fig. 2). For lower concentration of SDF-1α (10 ng/mL) there was no significant difference between the groups.

**Fig. 3:**
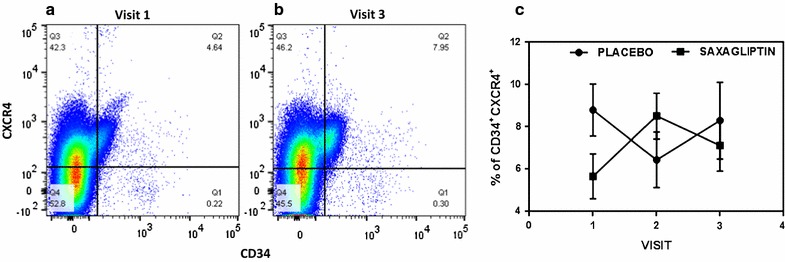
Representative image (**a** and **b**) indicating the CD34+ CXCR4+ expression in MNCs by flow cytometry. CD34+ CXCR4+ expression is higher for the saxagliptin group at visit 3 than visit 1 (7.95 and 4.64%, respectively). **c** Double positivity for CD34 and CXCR4 along the visits for placebo and saxagliptin groups (p < 0.01)

**Fig. 4:**
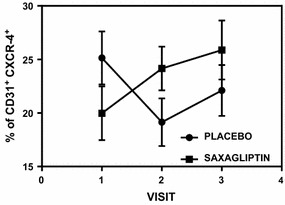
CD31+ CXCR4+ expression in MNCs assessed by flow cytometry. Double positivity of CD31 and CXCR4 is higher for saxagliptin group at visit 2 and visit 3 in comparison to placebo group (p < 0.01)

The functional improvement of CD34+ cells described above (Fig. 2) reflects the higher percentage of CD34+ CXCR4+ expression (visit 1 = 5.6% ± 1.0; visit 2 = 8.5% ± 1.1; visit 3 = 7.1% ± 1.2) present in the MNCs after saxagliptin treatment. Therefore, a 12-week trial of saxagliptin therapy led to a significant increase in circulating EPCs expressing CXCR4 (measured by flow cytometry; p < 0.01) facilitating the migration process promoted by SDF-1α (Fig. 3). Remarkably, there was a dramatic increase in CD34+ CXCR4+ cell numbers particularly at visit 2 (at 6 weeks of treatment), in spite of lower levels in saxagliptin group at the onset or visit 1. Similarly, in Fig. 4, we noted increased number of circulating endothelial cells, identified as CD31+ cells on flow-cytometry, across visits 2 and 3.

The number of CD34+ cells present in the MNCs pool is relatively low in patients with diabetes. Thus, in this study the number of CD34+ cells obtained for isolation of mRNA was not always sufficient or adequate in order to obtain confident gene expression analysis. As an alternative we looked at mRNA gene expression from CD34− cells (Mononuclear cell population (MNC) minus CD34+ cells). CD34− cell population is largely reflective of the MNC population (minus approx. 1% CD34+ cells).

**Fig. 5:**
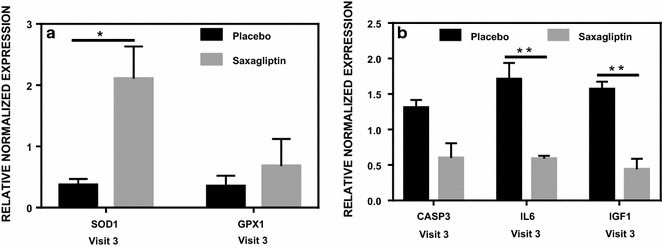
Effect of saxagliptin on gene expression of CD34+ (**a**) and CD34− (**b**) cells. A low number of CD34+ cells obtained resulted in a reduced amount of mRNA affecting the number of samples suitable for analysis. The genes affected by saxagliptin treatment were: SOD1 (n = 11), GPX1 (n = 11), CASP3 (n = 10), IL6 (n = 13), IGF1 (n = 7). *p < 0.05; **p < 0.01. Results are relative to visit 1 (control)

Gene expression analysis was performed for antioxidants (SOD1, SOD2, GPX1, CAT), apoptosis (BCL-2, CDKN1A, TP53, CASP-3), endothelial function (VEGFA, VEGFR2, EDN-1, eNOS, IGF1), cell chemotaxis (SDF-1α, CXCR4), inflammation (IL-6, TNFα), progenitor marker (CD34) and endothelial lineage cell surface markers (PECAM1). For CD34+ cells, upregulation (2.1-fold) was observed for SOD1 (p < 0.05); while for CD34− cells, downregulation was observed for IL-6 (approximately 40%) and IGF-1 (approximately 60%) (p < 0.01) (Fig. 5). There was also a trend for upregulation (p = 0.1893) for GPX1 expression for CD34+ cells. Both GPX1 and SOD1 are cytosolic anti-oxidants, which appear to increase in expression post saxagliptin therapy. For CD34− cells a trend for downregulation was noted for caspase 3 (p = 0.08) (Fig. 5), a well-known pro-apoptotic gene.

### Secondary outcomes: clinical

The study population was representative of subjects with uncontrolled type 2 diabetes, but with no preexisting macro-vascular complications. All adverse effects that occurred throughout the duration of the study were either not related to the study medication and design, or fell within the expected side effects profile for saxagliptin.

Table 2 shows blood biochemistries and arterial stiffness measures across the three visits.

**Table 2:** Blood biochemistry and arterial stiffness before and after saxagliptin treatment

	Visit 1	Visit 2	Visit 3	p-value*
***Blood biochemistries***				
*Glucose*				
Placebo	114.8 ± 5.3	113.8 ± 5.5	112.1 ± 4.8	0.233
Saxagliptin	125.9 ± 7.8	113.5 ± 7.6	117.1 ± 5.8	
*BUN*				
Placebo	14.0 ± 0.9	13.0 ± 0.8	13.7 ± 0.7	0.205
Saxagliptin	13.1 ± 0.7	13.9 ± 0.8	13.5 ± 0.8	
*Serum creatinine*				
Placebo	1.2 ± 0.3	1.0 ± 0.1	0.9 ± 0.1	0.118
Saxagliptin	0.9 ± 0.0	0.9 ± 0.0	0.8 ± 0.0	
*eGFR*				
Placebo	93.7 ± 3.6	93.0 ± 3.8	92.7 ± 3.9	0.357
Saxagliptin	98.3 ± 3.1	93.4 ± 2.7	26.9 ± 2.9	
*Cholesterol*				
Placebo	174.5 ± 9.9	166.0 ± 7.1	164.3 ± 7.0	0.299
Saxagliptin	170.1 ± 8.1	168.8 ± 6.6	171.8 ± 8.5	
*Triglycerides*				
Placebo	106.2 ± 7.3	112.0 ± 10.0	107.8 ± 7.3	0.972
Saxagliptin	122.3 ± 13.7	126.5 ± 13.1	121.7 ± 11.3	
*LDL/HDL*				
Placebo	2.3 ± 0.2	2.1 ± 0.2	2.1 ± 0.2	0.160
Saxagliptin	1.8 ± 0.1	1.8 ± 0.1	1.8 ± 0.2	
*HbA1C*				
Placebo	6.6 ± 0.1	6.6 ± 0.1	6.5 ± 0.1	0.164
Saxagliptin	7.0 ± 0.2	6.8 ± 0.2	6.7 ± 0.2	
*C-reactive protein*				
Placebo	2.4 ± 0.6	2.9 ± 0.8	2.9 ± 0.7	0.156
Saxagliptin	2.8 ± 0.5	2.7 ± 0.4	2.4 ± 0.4	
*IL-6*				
Placebo	2.7 ± 0.6	4.0 ± 0.7	4.3 ± 0.9	0.629
Saxagliptin	3.1 ± 0.4	3.9 ± 0.7	3.8 ± 0.8	
*TNF-α*				
Placebo	3.1 ± 0.9	2.8 ± 1.1	1.6 ± 0.2	0.213
Saxagliptin	1.7 ± 0.2	1.9 ± 0.2	2.9 ± 1.3	
*Leptin*				
Placebo	14.1 ± 2.1	13.1 ± 1.8	13.8 ± 2.4	0.409
Saxagliptin	19.4 ± 3.7	17.4 ± 2.8	20.4 ± 3.5	
*Adiponectin*				
Placebo	4.6 ± 0.6	4.9 ± 0.6	5.5 ± 0.6	0.010*
Saxagliptin	4.2 ± 0.6	4.9 ± 0.7	4.0 ± 0.5	
*GLP1* (*ELISA*)				
Placebo	271.9 ± 67.2	241.1 ± 63.8	295.7 ± 78.5	0.400
Saxagliptin	245.9 ± 59.0	245.3 ± 55.5	234.0 ± 58.7	
*SDF-1ɑ* (*ELISA*)				
Placebo	− 1.84 ± 0.27	− 1.83 ± 0.27	− 1.80 ± 0.27	0.245
Saxagliptin	− 1.99 ± 0.27	− 1.61 ± 0.27	− 1.87 ± 0.25	
***Arterial stiffness***				
*Diastolic blood pressure (radial)*				
Placebo	82.7 ± 1.8	82.4 ± 2.3	82.0 ± 2.0	0.3723
Saxagliptin	84.9 ± 1.5	84.3 ± 1.2	81.9 ± 1.1	
*Diastolic blood pressure (arterial)*				
Placebo	84.4 ± 1.4	83.4 ± 2.2	83.1 ± 2.0	0.568
Saxagliptin	85.4 ± 1.5	84.6 ± 1.1	82.8 ± 1.1	
*Systolic blood pressure (radial)*				
Placebo	131.8 ± 3.6	126.0 ± 4.3	134.0 ± 3.5	0.009*
Saxagliptin	132.7 ± 2.5	133.1 ± 1.8	127.7 ± 2.3	
*Systolic blood pressure (arterial)*				
Placebo	118.7 ± 3.0	130.0 ± 7.9	121.2 ± 3.3	0.061
Saxagliptin	121.8 ± 1.7	117.5 ± 2.2	122.6 ± 2.6	
*Augmentation index-75*				
Placebo	18.4 ± 2.4	26.0 ± 3.9	23.3 ± 2.3	0.037*
Saxagliptin	24.1 ± 2.1	22.5 ± 2.0	23.1 ± 2.1	

* p-values are for the treatment group by visit interaction in the mixed model. This indicates whether the treatment groups had different slopes over time

Actigraph energy monitor use was analyzed to account for any exercise or activity level difference between the placebo and the saxagliptin treatment group. Post Actigraph analysis, we noted no difference between the two groups for the amount of hours per day spent in any level of physical activity intensity (sedentary, light, moderate, moderate-to-vigorous, or vigorous).

**Fig. 6:**
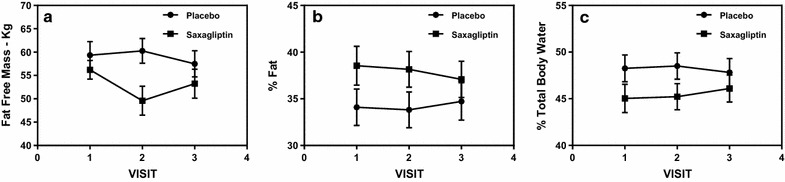
**a** Fat free mass (kg) show that the saxagliptin group had a sharp decline after visit 1, but then a rise after visit 2, and the placebo group remained relatively stable (0.072). **b**  % Body Fat across visits 1–3. Saxagliptin patients had a decline from visit 1 to 3, whereas the control group increased at visit 3 (p = 0.079). **c** % total body water remained relatively stable for the placebo group, but increase in saxagliptin (p = 0.098)

Body composition measures showed no statistically significant changes. A high correlative effect was seen in the Tanita body composition scale measures for fat free mass (FFM, p = 0.07) and percent body fat, (p = 0.08). Mean FFM, by visit, drops more from visit 1 to visit 2 in the saxagliptin group, than the control group at a trend level significance (Fig. 6a). Percent body fat, while being higher on average in the saxagliptin group than the placebo group, showed a decline after visit 2. Conversely, the placebo group had an increase after visit 2 (Fig. 6b). A trend was also noticed in total body water (TBW, p = 0.1), which was seen to decline from visit 1 to visit 2, and then a rise again at visit 3 in the saxagliptin group (Fig. 6c). In the control group, however, TBW remains stable until visit 2, when it begins to fall for visit 3.

**Fig. 7:**
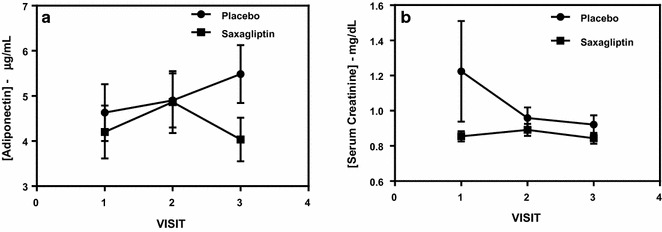
**a** Shows adiponectin values from visit 1 to visit 3. Saxagliptin showed a decrease throughout the study, after visit 2, while the placebo group increased steadily from visit 1 to visit 3 (p = 0.01). **b** Shows serum creatinine values from visit 1 to visit 3. There is a steep decline from visit 1 to 2 in the placebo group, while levels remain stable in the saxagliptin group (p = 0.12)

Venous blood biochemistries were gathered both through Labcorp of America and through serum ELISA. Both standard of care, and research values were collected. In the Labcorp values, only adiponectin (p = 0.01) was statistically significant across the saxagliptin and placebo groups. The placebo group had a steady increase across visit 1 through 3, but the saxagliptin saw an overall stable value of adiponectin (Fig. 7a). Serum creatinine remained relatively stable throughout the study in the saxagliptin group, but showed a drastic decrease after visit 1 in the placebo group, before the values leveled out (p = 0.12, Fig. 7b). There was no correlating significant or trend level significant noticed in eGFR (p = 0.36), or BUN. There were no statistically significant changes between groups in either GLP1 or SDF-1α, performed by ELISA.

For basal metabolic rate, there were no values that showed any statistically significance, when compared to the control group for any of the parameters.

**Fig. 8:**
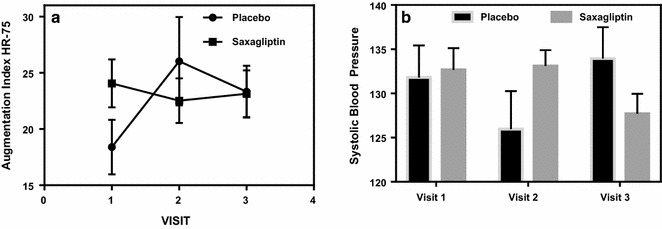
**a** Shows the arterial stiffness parameter, augmentation index adjusted for a heart rate of 75. Saxagliptin subjects remained stable across visit 1 through 3, whereas the control subjects had an increase in arterial stiffness (p = 0.04). **b** Shows radial systolic blood pressure in saxagliptin, compared to Placebo, across the three visits. Saxagliptin blood pressure remains stable across visits 1 and 2, but then drops in visit 3. In the placebo group, blood pressure drops at visit 2, but then rises dramatically by visit 3 (p = 0.009)

When analyzing the arterial stiffness, augmentation index that was adjusted for a heart rate of 75 (AI-75), was found to be statistically significant (p = 0.04), with the values for the control group increasing across visit 1 through 3. Saxagliptin subjects have a slight decline across the three visits (Fig. 8a). Systolic blood pressure, measured radially, was found to be statistically significant (Fig. 8b; p = 0.009). There was a trend level significant reduction for systolic blood pressure, measured arterially (Table 2; p = 0.061), however not all subjects had PWV measured, therefore lack of statistical difference (though there was a trend) between the groups may not be a true reflection.

In general, it was noted that adding HbA1c as a time-varying covariate had little impact on the results.

## Discussion

### Primary outcomes: cellular

We have defined EPCs as CD34+ cells. Werner et al. defined EPCs as CD34/KDR positive cells [27]. However, if an investigator uses two markers to isolate EPCs from mononuclear cells (MNCs) rather than one, the percentage of positive cells drops by almost 10-fold. In this study, only 60–80 mls of whole blood such a drop makes a cell based clinical trial difficult to interpret. Therefore, we have used only one marker, CD34+ve, so that we get consistently close to 1% population of MNC, as EPCs.

In this study we show for the first time that saxagliptin, in addition to metformin, positively modulates CD34+ EPCs, as a marker of vascular endothelial function, in early onset type 2 diabetes subjects who have no overt cardiovascular complications. Vascular complications are common in subjects with diabetes and a reduced number of EPCs in these subjects can predict early onset of vascular complications [28–31]. Therefore, in order to assess cardiovascular risk using a cell as a biomarker rather than serum biochemistry, it is important to investigate the number and functionality of endothelial progenitors and whether these cells can adequately promote or help with endothelial repair and angiogenesis [32]. Metformin, a very common medication for diabetes, has been shown to contribute to the improvement of EPCs in both type 1 and type 2 diabetes on its own and similarly, saxagliptin has been shown to increase EPC number and function [17, 22, 33]. Curiously, no differences in EPCs number were observed between the metformin and saxagliptin groups [17]. However, here we have demonstrated an additional improvement of EPCs by adding saxagliptin to on-going metformin treatment.

Based on the CFU-Hills’s colony count and migratory response to SDF-1α results (Figs. 1, 2, respectively), we demonstrated that saxagliptin enhances EPCs functionality. Considering that CFU-Hill’s colonies number is inversely related to Framingham risk score, these results also indicate a decrease in CVD risk promoted by the use of saxagliptin and metformin combination, in addition to exercise.

Our data also indicated a trend for an increase of CD34+ cell number for the saxagliptin group. On the other hand, other results recently published showed a significant increase of EPCs number (CD34+ CD133+ KDR+) when patients were subjected to either saxagliptin or metformin alone [17, 22, 33]. These results indicate that the combination of these two drugs may not promote additional increase in number of CD34+ cells in the periphery blood. It is possible that the subjects may have reached the maximum threshold already by using metformin. Metformin has a dual effect by simultaneously increasing VEGFA and reducing both CXCL10 and TIMP1 in CD34+ cells in a model of the diabetic state combined with hypoxia, and also shows a proangiogenic activity [34, 35].

Although we did not observe a statistically significant increase in CD34+ cells, the higher percentage of CD34+ CXCR4+ (double positive cell) for patients undergoing saxagliptin treatment was confirmed by our flow cytometry results (Fig. 3). We also noted increased CD31 positive cells that were CXCR4 positive (Fig. 4). CXCR4 is the receptor to SDF1α ligand. Similarly, other investigators have shown increase in the number of CD31+ cells as a positive outcome marker for cardiovascular risk assessment [36, 37]. It was noted that both CD34+ cells (a progenitor endothelial cell pool) and CD31+ cells (a mature circulating endothelial cell pool) had increased in double positivity with SDF1α receptor CXCR4 indicating positive effect of saxagliptin on endothelial lineage cells both immature and mature. It has been reported that CD34+ cells from patients with type 2 diabetes have defective chemotaxis to SDF-1α resulting in reduced vasculogenic potential [38]. Here, we showed that migratory response to SDF-1α of CD34+ cells is improved by saxagliptin. These results corroborate with the fact that CD34+ cells expressed more CXCR4 receptor and thereby facilitating the responsiveness to the SDF-1α ligand. Altogether, these results indicate that colony formation ability and mobilization of EPCs can be improved in patients with type 2 diabetes subjected to saxagliptin treatment. Similarly CD31+/CXCR4+ cell number also increased post saxagliptin therapy. While saxagliptin and metformin promoted similar beneficial effects on endothelial function when used separately, our results indicate a synergistic positive effect of saxagliptin to the entire endothelial lineage [17].

Effects of sitagliptin, another DPP-4 inhibitor, on EPCs have been previously reported [12, 13]. In type 2 diabetic patients, sitagliptin promoted increase in circulating EPCs followed by upregulation of SDF-1α [12]. In a mouse model, SDF-1α was also increased and in addition to increased EPCs number those results indicated that sitagliptin has a great potential as a promoter of neovascularization [13]. However in our study, saxagliptin therapy did not show an increase in SDF-1α plasma levels (measured by ELISA). There are still no data showing the minimum period of time for administration of saxagliptin in order to demonstrate an increase in SDF-1α levels. Therefore, our results could be secondary to a short-term intervention of 12 weeks and it is likely that plasma levels did not show increase as optimal time for detecting a statistical difference between placebo and saxagliptin. Interestingly, SDF-1α mRNA expression of EPCs was also not upregulated, in spite of increased CXCR4− expressing CD34+ cell number. In fact, there was a trend towards down-regulation of SDF-1α in the mRNA expression from cells that were exposed to saxagliptin, which may indicate that saxagliptin is rescuing the cells from damage and dysfunction, as SDF-1α is a factor that is primarily produced from damaged cells rather than healthy cells. Therefore the gene expression of SDF-1α ligand in healthy EPCs may actually decrease [29, 31]. The lack of effects of saxagliptin on SDF-1α plasma levels needs to be confirmed in larger and longer outcome studies.

Regarding gene expression, we looked at pathways involving inflammation, oxidative stress, apoptosis, EPC chemotaxis pathways and endothelial function markers, in both CD34+ and CD34− cells obtained post magnetic column separation of MNCs. The mRNA expression results that stand out (statistically significant) are the antioxidants such as SOD1 (superoxide dismutase) and GPX1 (glutathione peroxidase) with clear upregulation, post Saxagliptin exposure, compared to placebo. This is concurrent with reduced inflammatory and apoptosis cascade markers such as IL6 and Caspase-3 mRNA expressions in CD34− cells indicating an overall reduction in inflammation and apoptosis pathway, which could be secondary to upregulation in antioxidants. This is particularly interesting as there is sufficient evidence from our previous work, to suggest significant patho-physiological role of ROS (anti-oxidant expression may increase in response to increased intracellular ROS presence) in not only EPCs but also in MSCs in a setting of hyperglycemia, as seen in diabetes [10, 39]. The decrease of IGF-1 mRNA expression in CD34− cells in the saxagliptin intervention group compared to placebo may indicate reduced insulin resistance at the levels of MNCs, which also supports reduction of cellular inflammation.

### Secondary outcomes: clinical

All subjects were on a stable dose of metformin (1–2 grams/day) for 3 months or greater prior to enrollment. Subjects were instructed to engage in 150 min of moderate intensity physical activity per week, as per the American Diabetes Association guidelines, prior to being randomized into either the saxagliptin or placebo group. The Actigraph energy monitor data analysis showed no difference in activity across all exercise intensity levels between saxagliptin and placebo. This indicates that no changes for any outcome measure in the saxagliptin group can be attributed to exercise alone.

Saxagliptin is a weight neutral medication, and thus it was not expected that we would find a difference between groups in the weight [40]. There also was no change in waist or hip circumference measurements, which is consistent with other studies involving saxagliptin and linagliptin, although these studies did not have concomitant metformin therapy [17, 41]. Previous studies involving mice have shown that treatment with DPP-4 inhibitors in hyperglycemic, obese mice resulted in reductions in adiposity, both in body fat percentage, and abdominal fat mass [42]. This was attributed to an increase in energy expenditure, which was measured via monitoring metabolic rate and food intake. Our research subjects taking saxagliptin had reductions in both fat free mass (kg) and percent fat as compared to placebo, however, there was no difference between groups in resting energy expenditure values or exercise levels. The changes in fat mass, therefore, could likely be attributed to saxagliptin. The reduction in fat mass could be secondary to increased fat oxidation in lean and obese conditions [43]. Total body water showed a rise from visit 2 to 3 in the saxagliptin group, whereas the control group remained steady. One reason might be related to the increase of heart failure rate noticed in the SAVOR-TIMI trial, however this trial, unlike ours, involved a population with pre-existing CVD [44]. Interestingly, reduction in fat mass and increase in TBW would keep the weight of the subjects neutral as seen in most trials with DPP4 inhibitors, however this may not be clinically relevant in subjects with no overt heart failure [16].

Previous studies investigating DPP-IV inhibitor therapy, mainly sitagliptin and vildagliptin, in a type 2 diabetes population found that there was no significant reduction in HbA1C values with treatment [45]. This is consistent with our results, upon comparing the saxagliptin and placebo groups. In the saxagliptin group HbA1c decreased by 0.3% (less than expected), and the baseline HbA1c value was higher than placebo group. The difference in the baseline HbA1c value between groups and the little HbA1c reduction on the saxagliptin group is unlikely to explain the positive effect of saxagliptin on CD34+ EPC primarily based on HbA1C changes. Therefore, the positive changes that we see such as in migration response of EPCs to SDF1a and flow cytometry is unlikely to be secondary to improvement in glycemic indices. One study conducted by Li et al. found a significant improvement in HbA1C with saxagliptin therapy over 12 weeks, but these subjects were naïve to anti-diabetic therapy prior to entering the study [17]. Blood biochemistries, however, did find a significant difference both in adiponectin and serum creatinine between saxagliptin and placebo. While the meta-analysis conducted by Liu et al. found that treatment with DPP-4 inhibitors resulted in an increase in adiponectin, as compared to control, our results showed the opposite [46]. A reason for this may be that in our study we actually noticed a reduction in percent fat, and overall fat mass. We however did not see a decrease in leptin values.

We noted a stable serum creatinine in the saxagliptin group compared to the placebo group. The eGFR was not statistically significant (p = 0.36), which may be because the eGFRs for the placebo group were initially lower and remained stable throughout the study. On the other hand, despite creatinine starting higher, it improved significantly in the 1st phase of the study. Therefore, improvement in plasma creatinine may be an acute effect rather than persistent or chronic effect. This maybe an interesting finding considering the fact that diabetes related kidney disease is a progressive disease. This could be explained by the fact that saxagliptin most likely has a renal protective effect. A higher percentage of CD34+ CXCR4+ cells may which help by homing-in on the kidney vasculature to repair the endothelial dysfunction present and thereby help prevent progressive kidney damage [47, 48].

Arterial stiffness, is a measure of compliance of one’s arteries, and their ability to constrict and dilate in response to blood pressure [49–51]. It is measured non-invasively by assessing PWV) and PWA, and has been noted to increase naturally with age [49]. PWV is measured as a velocity in m/s. Higher values of AP, AI, AI-75, and PWV are correlated to higher levels of arterial stiffness. PWV, in addition to PWA measures such as blood pressure and augmentation index, have been found to be a predictor of increased CVD risk in the general population, and especially in those at an increased risk, such as patients with type 2 diabetes [49, 51]. Arterial stiffness, being a direct measure of the radial, carotid and femoral arteries, would be expected to change with significant alterations to the endothelium.

Our study shows a reduction in arterial stiffness in the saxagliptin group, as seen through a reduction in AI-75. This was also seen with other DPP-4 inhibitors, sitagliptin and vildagliptin, which resulted in a reduction in AI-75 [45]. Arterial stiffness, as measured via AI-75, is a strong predictor of CVD in Type 2 Diabetes [52, 53]. The reduction in AI-75 may be attributed to a multi-platform effect. DPP-4 inhibitors cause an increase in systemic incretin levels, which can cause a relaxation of the arteries via nitric oxide (NO) [54, 55]. This could be attributed to a reduction in arterial stiffness. Also, the increase in CFU and higher percentage of CD34+ CXCR4+ that was reported in our cellular analysis may indicate that EPCs are having a regenerative effect on the subjects’ arteries across the 12-week time-period. Finally, DPP-4 inhibitors help patients achieve a better level of glycemic control, and, as observed from our body composition results, a decrease in both percent fat and fat free mass. This decline in adiposity may also contribute to the improvement (reduced PWA) in arterial stiffness.

ELISA was performed on patient serum samples to check for the presence of GLP1, and SDF-1α, in order to confirm that the saxagliptin was effectively blocking DPP-4 in the body. We were anticipating an increase in serum GLP1and SDF-1α in the saxagliptin group, indicating that DPP-4 was successfully inhibited. However, our results found no statistical significance between saxagliptin and placebo groups. It is likely that metformin leads to a significant increase of GLP-1 as seen in obese non diabetic and type 2 diabetes subjects, therefore the addition of saxagliptin would not show any further appreciable changes [56, 57]. Alternatively, it is possible that the dose of 5 mg of saxagliptin or duration of only 12 weeks was not sufficient to show a difference, or increase in values.

### Limitations

Limitations of our study may include the relatively short duration of 12 week saxagliptin therapy, which may have been inadequate to see significant changes in certain clinical and cellular parameters. This may have been because of the small sample size, and due to the difficulty in obtaining all measures, in some patients. Further studies with a larger population and longer duration would be helpful to further explore and consolidate the mechanisms behind our findings.

## Conclusion

There appears to be a synergic effect of using saxagliptin (DPP-4 inhibitor) and metformin together, which promote functional improvement of circulating EPCs (defined as CD34+ cells), which subsequently improves metabolic parameters, renal function, arterial stiffness and systolic blood pressure.

We conclude that CD34+ cell function improves post saxagliptin therapy compared to a matched placebo in an early onset type 2 diabetes population that did not have any known cardiovascular disease or complications. We believe CD34+ cells can act as a valuable biomarker for assessment of endothelial function, in a setting of diabetes and can help provide valuable information using cellular data, to support or refute findings from other cardiovascular outcome trials in diabetes [44, 58–60].

## Additional files

**Additional file 1: Appendix S1.** Inclusion and exclusion criteria.

**Additional file 2: Appendix S2.** Blood biochemistries and arterial stiffness before and after saxa treatment.

## Abbreviations

AI: augmentation index
AI-75: augmentation index adjusted for a heart rate of 75
AP: augmentation pressure
AS: arterial stiffness
BCl-2: B-cell lymphoma 2
BMI: body mass index
BUN: blood urea nitrogen
CASP-3: caspase 3
CAT: catalase
CDKN1A: cyclin-dependent kinase inhibitor 1A
CD34: progenitor marker
CFU: colony forming units
CVD: cardiovascular disease
CXCR4: C-X-C motif chemokine receptor 4
DBP: diastolic blood pressure
DPP-4 inhibitors: dipeptidyl peptidase-4 inhibitors
EDN-1: endothelin 1
eGFR: estimated glomerular filtration rate
ELISA: enzyme-linked immunosorbent assay
eNOS: nitric oxide synthase 3 (endothelial cell)
EPCs: endothelial progenitor cells
FFM: fat free mass
GAPDH: glyceraldehyde-3-phosphate dehydrogenase
GLP1: glucagon like peptide 1
GPX1: glutathione peroxidase 1
HbA1C: hemoglobin A1C, glycated hemoglobin test
IGF1: insulin-like growth factor 1
IL-6: interleukin 6
kg: kilograms (weight)
lbs: pounds (weight)
MNCs: mononuclear cells
PECAM1: platelet and endothelial cell adhesion molecule 1
PWA: pulse wave analysis
PWV: pulse wave velocity
qRT-PCR: quantitative reverse transcriptase polymerase chain reaction
REE: resting energy expenditure
SBP: systolic blood pressure
SDF1α: stromal cell-derived factor-1α
SOD1, SOD2: super oxide dismutase 1 and 2
TBW: total body water
TNFα: tumor necrosis factor α
TP53: tumor protein p53
VEGF: vascular endothelial growth factor
VEGFA: vascular endothelial growth factor A
VEGFR2: vascular endothelial growth factor receptor 2
VO2: maximal oxygen consumption
18S: 18S ribosomal RNA
