# Supplementary material for: The synergistic effects of saxagliptin and metformin on CD34+ endothelial progenitor cells in early type 2 diabetes patients: a randomized clinical trial
Source: Cardiovasc Diabetol. 2018 May 3;17:65. doi: 10.1186/s12933-018-0709-9 (PMC5934787; doi:10.1186/s12933-018-0709-9)
Supplement: Supplementary file 1 — Additional file 1: Appendix S1. Inclusion and exclusion criteria. [file 12933_2018_709_MOESM1_ESM.docx]

Inclusion Criteria

- Signed Informed Consent
- Type 2 diabetes mellitus within the previous 8 years using criteria of the American Diabetes Association.
- Currently treated with no hypoglycemic agents other than a stable dose (>3 months) of metformin (≥1.0 to ≤2 grams daily).
- HbA1C between 6% and 9% (both inclusive)
- BMI 25-39.9 kg/m^2^ both inclusive.
- Men and women, 40 to 70 years of age.

Exclusion Criteria

- Type 1 diabetes mellitus
- History of diabetic ketoacidosis or hyperosmolar nonketotic coma
- Hemoglobinopathies with low hematocrit (hematocrit below normal limits that may impair exercise tolerance) or abnormal CBC
- History of pancreatitis, or cancer (except basal cell carcinoma)
- Previous coronary or cerebrovascular event within 6 months of screening or active or clinically significant coronary and/or peripheral vascular disease
- Statin use started (or dose change) in the last 3months,
- Daily use of oral or injectable anti-diabetic medication other than Metformin
- use of any form of steroid medication (oral, inhaled, injected or nasal) within the last 3 months
- Systolic BP> 140 mmHg and Diastolic BP> 90mmHg at screening visit.
- Active wounds or recent surgery within 3 months
- Inflammatory disease, or current use of anti-inflammatory drugs
- Untreated hyper/hypothyroidism
- Contraindications for moderate exercise
- Implanted devices (e.g., pacemakers) that may interact with Tanita scale
- Pre-existing liver disease and/or ALT and AST >2.5X’s UNL,serum creatinine levels ≥1.5 mg/dL for men, ≥1.4 mg/dL for women, estimated CrCl ≤50 mL/min)
- Triglycerides >400 mg/dL
- Subjects with a history of any serious hypersensitivity reaction to saxagliptin or DPP-4 inhibitor.
- WOCBP who are unwilling or unable to use an acceptable method to avoid pregnancy for the entire study period.
- Women who are pregnant or breastfeeding.
- Treatment with systemic cytochrome P450 3A4 (CYP 3A4) inhibitors
- Prisoners or subjects who are involuntarily incarcerated.
- Subjects who are compulsorily detained for treatment of either a psychiatric or physical (e.g., infectious disease) illness.
- Patients who are active smokers
- Patients who are pregnant, nursing women
- Post-menopausal women who are on hormone replacement therapy will be excluded. Patients on low dose oral contraceptives will be allowed to participate as these formulations contain lesser amount of estrogens.

*Eligibility criteria for this study have been carefully considered to ensure the safety of the study subjects and to ensure that the results of the study can be used. It is imperative that subjects fully meet all eligibility criteria.*
