# Supplementary material for: The synergistic effects of saxagliptin and metformin on CD34+ endothelial progenitor cells in early type 2 diabetes patients: a randomized clinical trial
Source: Cardiovasc Diabetol. 2018 May 3;17:65. doi: 10.1186/s12933-018-0709-9 (PMC5934787; doi:10.1186/s12933-018-0709-9)
Supplement: Supplementary file 2 — Additional file 2: Appendix S2. Blood biochemistries and arterial stiffness before and after saxa treatment. [file 12933_2018_709_MOESM2_ESM.docx]

| **Appendix S2 –Blood Biochemistries and Arterial Stiffness before and after Saxa treatment** | | | | | |  |  |
| --- | --- | --- | --- | --- | --- | --- | --- |
| ***Blood Biochemistries*** |  | **Visit 1** | **Visit 2** | **Visit 3** | **p-value** |  |  |
| **Glucose** | Placebo | 114.8 ± 5.3 | 113.8 ± 5.5 | 112.1 ± 4.8 | **0.233** |  |  |
|  | Saxagliptin | 125.9 ± 7.8 | 113.5 ± 7.6 | 117.1 ± 5.8 |  |  |  |
| **BUN** | Placebo | 14.0 ± 0.9 | 13.0 ± 0.8 | 13.7 ±0.7 | **0.205** |  |  |
|  | Saxagliptin | 13.1 ± 0.7 | 13.9 ± 0.8 | 13.5 ± 0.8 |  |  |  |
| **Serum Creatinine** | Placebo | 1.2 ± 0.3 | 1.0 ± 0.1 | 0.9 ± 0.1 | **0.118** |  |  |
|  | Saxagliptin | 0.9 ± 0.0 | 0.9 ± 0.0 | 0.8 ± 0.0 |  |  |  |
| **eGFR** | Placebo | 93.7 ± 3.6 | 93.0 ± 3.8 | 92.7 ± 3.9 | **0.357** |  |  |
|  | Saxagliptin | 98.3 ± 3.1 | 93.4 ± 2.7 | 26.9 ± 2.9 |  |  |  |
| **Cholesterol** | Placebo | 174.5 ± 9.9 | 166.0 ± 7.1 | 164.3 ± 7.0 | **0.299** |  |  |
|  | Saxagliptin | 170.1 ± 8.1 | 168.8 ± 6.6 | 171.8 ± 8.5 |  |  |  |
| **Triglycerides** | Placebo | 106.2 ± 7.3 | 112.0 ± 10.0 | 107.8 ± 7.3 | **0.972** |  |  |
|  | Saxagliptin | 122.3 ± 13.7 | 126.5 ± 13.1 | 121.7 ± 11.3 |  |  |  |
| **LDL / HDL** | Placebo | 2.3 ± 0.2 | 2.1 ± 0.2 | 2.1 ± 0.2 | **0.160** |  |  |
|  | Saxagliptin | 1.8 ± 0.1 | 1.8 ± 0.1 | 1.8 ± 0.2 |  |  |  |
| **HbA1C** | Placebo | 6.6 ± 0.1 | 6.6 ± 0.1 | 6.5 ± 0.1 | **0.164** |  |  |
|  | Saxagliptin | 7.0 ± 0.2 | 6.8 ± 0.2 | 6.7 ± 0.2 |  |  |  |
| **C-Reactive Protein** | Placebo | 2.4 ± 0.6 | 2.9 ± 0.8 | 2.9 ± 0.7 | **0.156** |  |  |
|  | Saxagliptin | 2.8 ± 0.5 | 2.7 ± 0.4 | 2.4 ± 0.4 |  |  |  |
| **IL-6** | Placebo | 2.7 ± 0.6 | 4.0 ± 0.7 | 4.3 ± 0.9 | **0.629** |  |  |
|  | Saxagliptin | 3.1 ± 0.4 | 3.9 ± 0.7 | 3.8 ± 0.8 |  |  |  |
| **TNF-α** | Placebo | 3.1 ± 0.9 | 2.8 ± 1.1 | 1.6 ± 0.2 | **0.213** |  |  |
|  | Saxagliptin | 1.7 ± 0.2 | 1.9 ± 0.2 | 2.9 ± 1.3 |  |  |  |
| **Leptin** | Placebo | 14.1 ± 2.1 | 13.1 ± 1.8 | 13.8 ± 2.4 | **0.409** |  |  |
|  | Saxagliptin | 19.4 ± 3.7 | 17.4 ± 2.8 | 20.4 ± 3.5 |  |  |  |
| **Adiponectin** | Placebo | 4.6 ± 0.6 | 4.9 ± 0.6 | 5.5 ± 0.6 | **0.010*** |  |  |
|  | Saxagliptin | 4.2 ± 0.6 | 4.9 ± 0.7 | 4.0 ± 0.5 |  |  |  |
| **GLP1 (ELISA)** | Placebo | 271.9 ± 67.2 | 241.1 ± 63.8 | 295.7 ±78.5 | **0.400** |  |  |
|  | Saxagliptin | 245.9 ± 59.0 | 245.3 ± 55.5 | 234.0 ± 58.7 |  |  |  |
| **SDF-1α (ELISA)** | Placebo | -1.84 ± 0.27 | -1.83 ± 0.27 | -1.80 ± 0.27 | **0.245** |  |  |
|  | Saxagliptin | -1.99 ± 0.27 | -1.61 ± 0.27 | -1.87 ± 0.25 |  |  |  |
| ***Arterial Stiffness*** |  | | | | |  |  |
| **Diastolic Blood Pressure (Radial)** | Placebo | 82.7 ± 1.8 | 82.4 ± 2.3 | 82.0 ± 2.0 | **0.3723** |  |  |
|  | Saxagliptin | 84.9 ± 1.5 | 84.3 ± 1.2 | 81.9 ± 1.1 |  |  |  |
| **Diastolic Blood Pressure (Arterial)** | Placebo | 84.4 ± 1.4 | 83.4 ± 2.2 | 83.1 ± 2.0 | **0.568** |  |  |
|  | Saxagliptin | 85.4 ± 1.5 | 84.6 ± 1.1 | 82.8 ± 1.1 |  |  |  |
| **Systolic Blood Pressure (Radial)** | Placebo | 131.8 ± 3.6 | 126.0 ± 4.3 | 134.0 ± 3.5 | **0.009*** |  |  |
|  | Saxagliptin | 132.7 ± 2.5 | 133.1 ± 1.8 | 127.7 ± 2.3 |  |  |  |
| **Systolic Blood Pressure (Arterial)** | Placebo | 118.7 ± 3.0 | 130.0 ± 7.9 | 121.2 ± 3.3 | **0.061** |  |  |
|  | Saxagliptin | 121.8 ± 1.7 | 117.5 ± 2.2 | 122.6 ± 2.6 |  |  |  |
| **Augmentation Index -75** | Placebo | 18.4 ± 2.4 | 26.0 ± 3.9 | 23.3 ± 2.3 | **0.037*** |  |  |
|  | Saxagliptin | 24.1 ± 2.1 | 22.5 ± 2.0 | 23.1 ± 2.1 |  |  |  |
